# Supplementary material for: Downregulated Expression of miR-200c-3p in Plasma Exosome as a Potential Biomarker in Takayasu’s Arteritis
Source: Int J Mol Sci. 2025 Mar 22;26(7):2881. doi: 10.3390/ijms26072881 (PMC11988894; doi:10.3390/ijms26072881)
Supplement: Supplementary file 1 [file ijms-26-02881-s001.zip › ijms-3418062-supplementary.pdf]

**Supplemental Figure S1.**

**Supplemental Figure S2.**

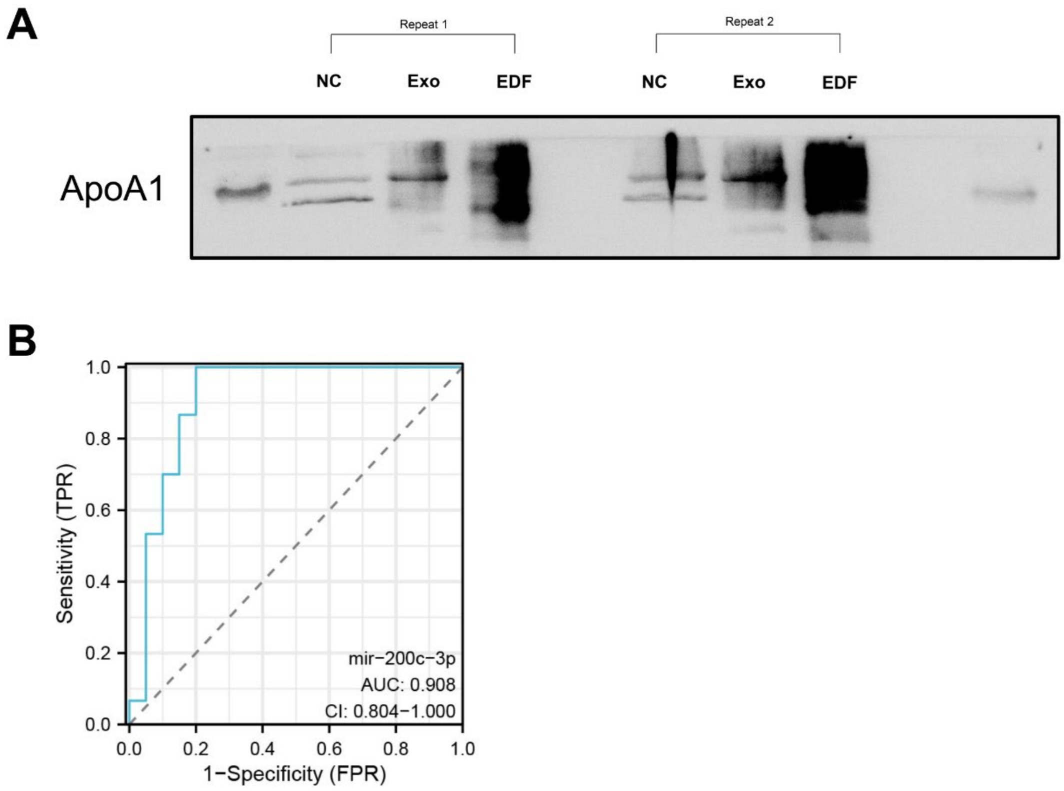


**Supplemental Figure S1.**

**A.** Representative Western blot showing ApoA1 expression in exosome preparations. Duplicate experiments show ApoA1 levels in negative control (NC), isolated exosomes (Exo), and the remaining cellular fraction after exosome isolation (Exosome-Depleted Fraction, EDF). **B.** ROC curve demonstrating the diagnostic potential of plasma exosomal miR-200c-3p for distinguishing treatment-naïve Takayasu's Arteritis (TAK) patients from healthy controls (HC). The analysis was performed using qPCR-determined miR-200c-3p levels in plasma exosomes from 30 treatment-naïve TAK patients and 20 healthy controls.

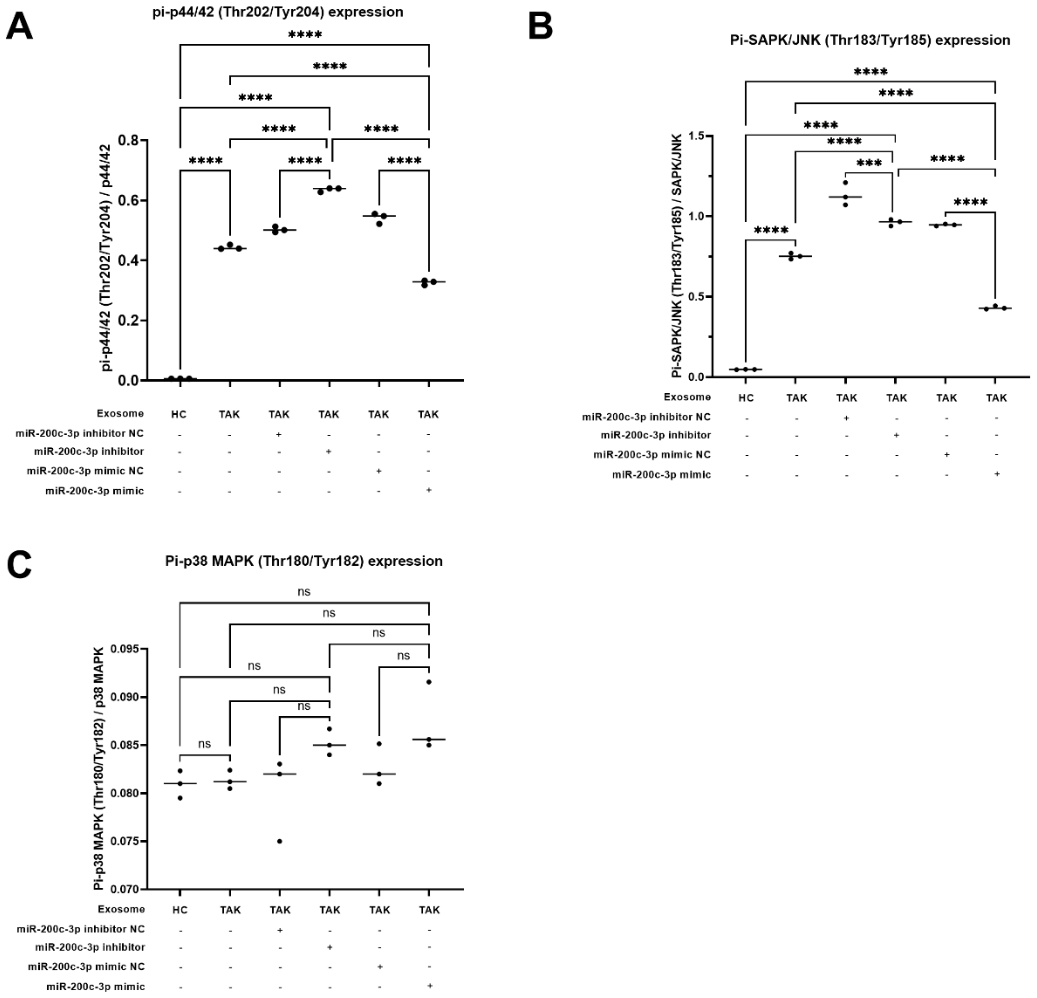


## Supplemental Figure S2.

**A.** Quantification of phosphorylated ERK1/2 (pi-p44/42 MAPK) protein levels in HAECs treated with plasma-derived extracellular vesicles (pEVs) from Takayasu Arteritis (TAK) patients/HC, with or without the addition of a miR-200c-3p mimic/inhibitor. **B.** Quantification of phosphorylated JNK (pi-SAPK/JNK) protein levels in HAECs treated with plasma-derived extracellular vesicles (pEVs) from Takayasu Arteritis (TAK) patients/HC, with or without the addition of a miR-200c-3p mimic/inhibitor. **C.** Quantification of phosphorylated p38 MAPK (pi-p38) protein levels in HAECs treated with plasma-derived extracellular vesicles (pEVs) from Takayasu Arteritis (TAK) patients/HC, with or without the addition of a miR-200c-3p mimic/inhibitor. Protein levels were determined by quantitative densitometry of Western blots using ImageJ. ns, non-significant, ***p<0.001, ****p<0.0001.
